# Implementation analysis of patient reported outcomes (PROs) in oncological routine care: an observational study protocol

**DOI:** 10.1186/s12955-019-1262-2

**Published:** 2020-01-02

**Authors:** Mirja Gianna Görlach, Theresa Schrage, Carsten Bokemeyer, Nicolaus Kröger, Volkmar Müller, Cordula Petersen, Christian Stephan Betz, Andreas Krüll, Holger Schulz, Christiane Bleich

**Affiliations:** 1 Department of Medical Psychology, University Medical Center Hamburg Eppendorf, Martinistraße 52, 20246 Hamburg, Germany; 2 II. Medical Clinic, Department for Oncology, Hematology, BMT with Section Pneumology, University Medical Center Hamburg Eppendorf, Martinistraße 52, 20246 Hamburg, Germany; 3 Department of Stem Cell Transplantation, University Medical Center Hamburg Eppendorf, Martinistraße 52, 20246 Hamburg, Germany; 4 Department of Gynecology, University Medical Center Hamburg Eppendorf, Martinistraße 52, 20246 Hamburg, Germany; 5 Department of Radiotherapy and Radiation Oncology, University Medical Center Hamburg Eppendorf, Martinistraße 52, 20246 Hamburg, Germany; 6 Department of Otolaryngology, University Medical Center Hamburg Eppendorf, Martinistraße 52, 20246 Hamburg, Germany

**Keywords:** PROs, HrQoL, Implementation, Psychooncology, eHealth, Cancer, Evaluation

## Abstract

**Background:**

The successful implementation of patient-reported outcomes (PROs) in clinical routine faces many challenges, first and foremost the lack of consideration thereof in the patient care process. The aim of this study will be to first identify relevant barriers and facilitators and then design suitable implementation strategies which will be evaluated to improve the effectiveness of a PRO measure assessment in inpatient and outpatient cancer routine care.

**Methods:**

During the preparation phase, interviews with oncological patients (*N* = 28) and medical staff (*N* = 4) as well as focus groups with medical staff (*N* = 18) across five different departments caring for cancer patients were conducted. On the basis of these, qualitative content analysis revealed relevant barriers and facilitators for implementation of PROs in cancer care. Subsequently, implementation strategies and a model of implementation were developed. In the study phase, implementation strategies will be evaluated based on nine different implementation outcomes in five different oncological clinics. Evaluation of the implementation process will take place during three months in each clinic and data will be conducted pre, while and post implementation of the PRO measure. Therefore a sample size of 60 participants of whom 30 staff members and 30 participants will be questioned using existing and newly developed implementation outcome evaluation instruments.

**Discussion:**

Key to improving the effectiveness of PRO assessment in the time-critical clinical environment is the utilization of easy-to-use, electronic PRO questionnaires directly linked to patients’ records thereby improving consideration of PROs in patient care. In order to validate the effectiveness of this implementation process further, an evaluation parallel to implementation following an observational study design with a mixed-methods approach will be conducted. This study could contribute to the development of adequate evaluation processes of implementation of PROs to foster sustainable integration of PRO measures into routine cancer care.

**Trial registration:**

This study was registered at Open Science Framework (https://osf.io/y7xce/).

## Contributions to the literature

- Research has shown that the successful implementation of PROs in cancer routine care faces many challenges, first and foremost the lack of consideration thereof in the patient care process.
- According to research and practice findings, inhibiting factors differ between oncological in- and outpatient clinics. Therefore, implementation strategies and process tailored to the individual needs of cancer units, patients and staff members have to be developed in order to facilitate implementation.
- This study contributes to the efforts of sustainably implementing PROs into cancer care through precise evaluation considering various implementation outcomes and measurements.

## Background

Today, patient reported outcomes (PROs) maintain an important role in patient centered care. PROs are self-assessment measures to collect information on health-related quality of life (HrQoL), physical discomfort or patient perceived health status [1]. Especially in chronic diseases PROs play an important role to generate data on the patient experience [2]. Therefore, research strongly suggests to implement PROs as a tool to improve the quality of patient care [2]. On the other hand it is surprising that the implementation of this information lags behind and that research is required in this respect [3]. HrQoL is one major dimension assessed through PRO measures in oncological care [4]. As cancer patients often experience physical and psychosocial consequences of their disease and its treatment, evaluation of HrQoL is important to get a full understanding of patient’s needs [5]. Interest in the use of HrQoL ratings in daily clinical practice has increased substantially [6]. However, successful implementation of PROs in clinical routine faces many challenges. Therefore, integration and use of results of PRO measures in oncological care is lacking [7].

Implementation is characterized as the use of strategies that serve to integrate and adapt an intervention into a specific setting [8]. Therefore, implementation research focuses on methods and strategies to understand and enhance successful integration of health care interventions [9]. In this context, Proctor et al. (2010) define implementation outcomes as “the effects of deliberate and purposive actions to implement new treatments, practices, and services”. Concluding, it is the aim of implementation research to provide sustainable and accepted implementation strategies for interventions to promote long-term use in routine care.

The evaluation of implementing health-related interventions in complex health care settings e.g. in the multidisciplinary setting of cancer care in a University Medical Center, is often poorly reported [10]. Implementation studies use diverse approaches and terminology to measure the success of implementation of interventions [11, 12]. However, to correctly interpret success or need for improvement of an intervention, it is key to be able to distinguish between poor efficacy of the intervention itself as a result of failure and unsuitable implementation strategies of the intervention [11]. Without detailed analysis of the implementation process, these sources of error are difficult to assign [13]. Therefore, it is important to also define and evaluate next to the intervention itself, the implementation process into clinical routine practice [14].

Many studies indicate that the use of PROs has been found to be useful, but there is often a lack of clear interpretation and structure for the application of the instrument in clinical routine [15]. From a clinician’s point of view, frequent barriers for implementation of PROs are lack of time, lack of training and support, and low personal confidence [16]. On the patient’s side, too burdensome interventions, e.g. too long or critical questions, can hinder effective implementation of PROs [17]. From an organizational level, resources and strategies for successful implementation are often missing [7]. Another problem arises when there is no adequate response by physicians e.g. to address critical PRO results [16]. Therefore, comprehensive research on inhibitory and beneficial factors for the use of PROs in clinical routine is important to facilitate the implementation process and to maintain the sustainability of PRO interventions in oncological care [17].

Proctor et al. (2011) propose eight dimensions following e.g. the RE-AIM Framework promoted by Glasgow (2007) to evaluate implementation of interventions in health care: Acceptability, Adoption, Appropriateness, Cost, Feasibility, Fidelity, Penetration and Sustainability [11, 18]. Implementation of interventions should be evaluated on these eight dimensions in order to gain precise information on the implementation process and to identify possible barriers. However, instruments evaluating implementation outcomes are lacking which leads to struggle in evaluating implementation processes satisfyingly [19]. Hence, important changes of implementations strategies cannot be made to further improve the implementation process.

Concluding, implementation science advises to consider certain factors that can substantially influence implementation efforts [20]. The purpose of our study is to identify beneficial and inhibiting conditions for clinicians and patients to use a PRO measure assessing health-related quality of life in cancer patients in clinical routine. On the basis of these findings, the PRO measure will be implemented into oncological routine care in a University Hospital in Germany. To ensure sustainable use of the PRO measure and its outcomes, the implementation will be evaluated following the dimensions proposed by Proctor et al. (2011) as well as the Consolidated Framework for Implementation Research (CFIR) [11, 12].

## Methods

### Design

To evaluate the implementation of the PRO measure, an observational study^1^ with a mixed method design will be conducted. The study contains of two phases and it is planned to combine qualitative and quantitative data in an exploratory mixed methods study design. Study participants will be recruited at five inpatient and outpatient clinics of the University Medical Centre Hamburg Eppendorf (II. Medical Clinic and Polyclinic, the Department of Stem Cell Transplantation, the Department of Gynecology, the Department of Radiotherapy and Radiation Oncology and the Department of Otolaryngology, where the PRO measure will be implemented and evaluated. Inclusion criteria for patients are inpatient or outpatient cancer treatment in one of the five clinics, sufficient language skills in German and no severe cognitive or verbal impairments in providing information and giving informed consent. The study received approval by the ethics committee of the medical association Hamburg (PV5636).

(For detailed outline of the study design see the enclosed Additional file 1 StaRI Checklist.)

#### Preparation phase

Aim of the preparation phase is to assess relevant barriers and facilitators for implementation of a PRO measure to assess HrQoL of cancer patients to prepare implementation in the study phase. For this purpose, interviews with oncological patients (*N* = 28) and oncologists (*N* = 4) were undertaken. To facilitate further discussion and exchange, five focus groups with oncologists, oncological nurses and psychologists were conducted. Patients as well as clinicians were asked to name possible barriers and facilitators for implementation of the PRO measure. The results were presented to a group of eight experts for discussion. Psychooncologists, oncologists, quality of life scientists, staff nurses, representatives of the quality management and a representative of a health insurance were present. On the grounds of the findings and of current state of research, the implementation process and implementation strategies were determined. Implementation strategies are: 1) a concise PRO measure (development of the questionnaire will be described elsewhere), 2) electronic input into a software with direct interface to patients’ records, 3) software and process training for medical staff and 4) guidance on pathways for further care. This preparation phase took place from December 2017 until April 2018.

#### Study phase

**Fig. 1:**
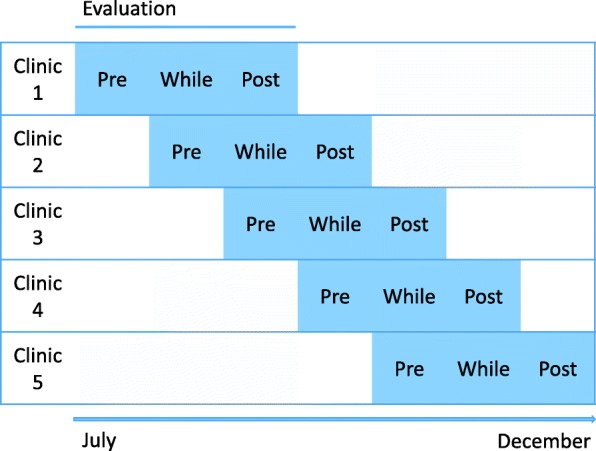
Implementation Process

In this study phase a PRO measure will be implemented into clinical routine practice at the University Medical Center Hamburg-Eppendorf at five oncological clinics. Gradually, one clinic after another will be included in the implementation. While implementation will take place, the implementation process will be evaluated in every clinic for three months: pre, while and post to first implementation of the PRO measure (see Fig. 1). Therefore, purposeful sampling will be used to ask medical staff member (i.e. nurses and doctors) to complete questionnaires assessing relevant implementation outcomes. For patients, purposeful sampling will be used by medical staff members to point out patients who are well enough to take part in semi structured interviews. Questionnaires will be presented in paper pencil format to medical staff members, interviews will be conducted by trained research assistants following semi structured interview guidelines. Additionally, one nurse and one physician of each clinic will be asked further questions concerning implementation outcomes in a semi structured interview. Furthermore, statistics on usage and response to the PRO measure will be retrieved from the electronic patient documentation system of the clinics. A pilot run will be conducted. Staff members of the University Medical Centre will be asked to give their impressions by using the thinking out loud technique, in order to assess the comprehensibility and feasibility of the evaluation questionnaires. The statistical survey will start at the end of July 2019 and end in December 2019.

### Cooperation partners

Recruitment of patients in study phase I will be carried out in cooperation with the II. Medical Clinic and Polyclinic, the Department of Stem Cell Transplantation, the Department of Gynecology, the Department of Radiotherapy and Radiation Oncology and the Department of Otolaryngology.

### Recruitment and procedure

#### Preparation phase

Potential patients to be questioned were pointed out by staff. The appointed patients were asked to participate and to give a written consent and were interviewed by scientific staff. Interviews with oncological patients (*N* = 28) and oncologists (*N* = 4) were undertaken. To facilitate further discussion and exchange, five focus groups with oncologists, oncological nurses and psychologists were conducted. Patients as well as clinicians were asked to name possible barriers and facilitators for implementation of the PRO measure. The results were presented to a group of eight experts for discussion.

#### Study phase

A random sample of eligible patients to be questioned will be pointed out by staff. Cross-sectional samples of patients as well as longitudinal samples of medical staff members will be questioned at three different times: pre, while and post implementation process. Unlike patients, same staff members will be questioned pre, while and post implementation. Regarding fluctuation of patients during evaluation of the implementation, different patients will be questioned while and post implementation process.

### Patient involvement

All three phases of the evaluation of the implementation will take into account appraisal of patients. Patients nor clinicians will be involved in the conception of the study.

### Measurements and outcomes

#### Preparation phase

A semi structured interview guide was developed based on Helfferich (2009) asking one main question concerning possible barriers and facilitators of implementation of a PRO measure in routine care [21]. Focus groups were carried out following a focus group guide referring to Barbour (2014) including the same main question as the interview guide [22].

#### Study phase

Implementation of the PRO measure will be evaluated based on the dimensions proposed by Proctor et al. (2011) [11]. Additionally, next to sociodemographic data the perceived benefit of the implementation of the PRO measure will be assessed. Different implementation outcome dimensions will be assessed at three stages of implementation: pre, while and post (see Table 1). “Acceptability” will be assessed using a German translation of the Acceptability E-Scale [23]) while as well as post implementation of the PRO measure. The 6-item questionnaire will be translated into German following the TRAPD protocol [24]. “Adoption” will be assessed pre and post implementation using the German translation of the Organizational Readiness for Implementing Change (ORIC) questionnaire [25]. “Appropriateness” will be assessed while and post implementation using the relevance scale of a German translation Workshop Evaluation Form (TCU Weval) questionnaire [26]. The items will be translated into German following the TRAPD protocol [24]. “Feasibility” will be assessed pre and post implementation using the program support scale of the Weval questionnaire [26]. The items will be translated into German following the TRAPD protocol [24]. “Cost” will be assessed pre implementation by one question on the expected time taken to record information and address problems according to the PRO measure and post implementation by one question about the time it actually takes for staff members to record information and address possible problems. “Fidelity” and “Penetration” will be assessed while and post implementation using one question for each dimension in a short survey as well as field notes taken by scientific staff members as well as statistical reports of clinical records. “Sustainability” will be assessed while and post implementation using two questions on the use of the PRO measure as well as through statistical reports of clinical records and field notes. “Benefit” will be assessed asking patients while and post implementation with one question about the perceived benefit of the PRO measure for the treatment of the patient. Staff members will be questioned on the “Benefit” by one question pre implementation on the expected benefit of the PRO measure and post implementation on the actual benefit for the treatment of patients of the PRO measure.

**Table 1:** Evaluation Process

Evaluation Step	Respondents	Instrument	Outcome
Pre	Staff Members	ORIC	Adoption
		WEVAL	Feasibility
		Questionnaire	Cost Benefit
While	Patients	Acceptability E-Scale	Acceptability
		WEVAL	Appropriateness
		Questionnaire	Benefit Sustainability
	Staff Members	Acceptability E-Scale	Acceptability
		WEVAL	Appropriateness
		Field Notes, statistical reports of clinical records, Questionnaire	Sustainability Fidelity Penetration
Post	Patients	Acceptability E-Scale	Acceptability
		WEVAL	Appropriateness
		Questionnaire	Benefit Sustainability
	Staff Members	ORIC	Adoption
		WEVAL	Feasibility
		Acceptability E-Scale	Acceptability
		WEVAL	Appropriateness
		Field Notes, statistical reports of clinical records, Questionnaire	Sustainability Fidelity Penetration
		Questionnaire	Cost Benefit

### Data analysis

#### Preparation phase

Interviews, focus groups and expert discussion were carried out by scientific staff, recorded and afterwards transcribed by staff members. The qualitative data was structured via MAXQDA 10 and analyzed using qualitative content analysis based on Mayring [27]. Within the procedure of analyzing the data, deductive-inductive category application was used: deductive main-categories (generated through literature research) and inductive sub-categories (derived from text analysis). Quality criteria to be examined for the qualitative content analysis were e.g. interrater reliability and communicative validation.

#### Study Phase

For quantitative data originated in the study phase, analyses of variance will be computed to compare the three different stages during implementation in the five clinics (SPSS Vers. 25). Missing data will be imputed using the expectation–maximization algorithm [28]. Transformations of data will only be applied, if data structure requires so (i.e. non normality of residuals).

### Sample size and power

Power calculations according to sample size calculations by Viechtbauer et al. (2015) [29] for this pilot evaluation suggest *N* = 59 with a confidence level of 95% and a low probability of the problem to occur of π = 0.05 in total. We therefore chose a number of *N* = 60 in total, *n* = 12 for every clinic participating which leads to *n* = 6 patients and *n* = 6 staff members of whom *n* = 3 nurses and *n* = 3 doctors in each clinic.

### Ethics and dissemination

The medical ethics committee of the Medical Chamber of Hamburg reviewed and approved the study protocol (date: 23 October 2017, number: PV5636). With this project it is intended to evaluate implementation of PROs into oncological routine care and to improve psychosocial care for cancer patients. Patients and health care professionals will be asked to participate by joining focus groups and interviews and by filling in questionnaires. Risks or disadvantages on the patient’s side are not expected. Written survey as a method does not involve direct intervention in medical procedures. A written informed consent is mandatory for participation in the study for patients as well as staff members. Patients participating in the study will be informed about voluntariness of participation and the possibility to refuse or discontinue participation at any time without any negative consequences. For further questions concerning the study, contact details of study assistants will be displayed.

The project duration is 36 months. The study was initiated in May 2016. Within the first 7 months, extensive preparatory work was carried out. The recruitment of participants for the preparation phase started in November 2016 and will begin at the end of July 2019 for the study phase. Completion of data collection is planned at the end of December 2019. Data entry, management and analysis as well as the publication of the findings in peer-reviewed journals and at conferences will take place continuously.

## Discussion

The use of PROs in oncological routine care to assess HrQoL in cancer patients can improve health care by assessing relevant symptoms and burdens in HrQoL. Furthermore, instant reaction to critical outcomes on HrQoL measures by clinicians is crucial in order to facilitate optimal treatment of cancer patients. However, implementation of PROs is often unsuccessful and unsustainable. One reason for this could be insufficient evaluation of the implementation process in order to detect possible barriers and facilitators to implementation and respond to those during or after evaluation process. We consider the chosen theoretical models for this study as reasonable and the evaluation tools sufficient in reliability and validity. Qualitative methods in this study are reasonable and chosen approach is feasible. Therefore, this study could contribute to the development of adequate evaluation processes of implementation of PROs to foster sustainable integration of PRO measures into routine cancer care.

## Supplementary information

**Additional file 1:** Standards for Reporting Implementation Studies: the StaRI checklist for completion

## Data Availability

Not applicable

## Abbreviations

CFIR: Consolidated Framework for Implementation Research
HrQoL: Health-related quality of life
PRO’s: Patient reported outcomes

## Footnotes

1 Registration of this study at Open Science Framework (https://osf.io/y7xce/).
