# Supplementary material for: Implementation analysis of patient reported outcomes (PROs) in oncological routine care: an observational study protocol
Source: Health Qual Life Outcomes. 2020 Jan 2;18:3. doi: 10.1186/s12955-019-1262-2 (PMC6941251; doi:10.1186/s12955-019-1262-2)
Supplement: Supplementary file 1 — Additional file 1: Standards for Reporting Implementation Studies: the StaRI checklist for completion [file 12955_2019_1262_MOESM1_ESM.docx]

**
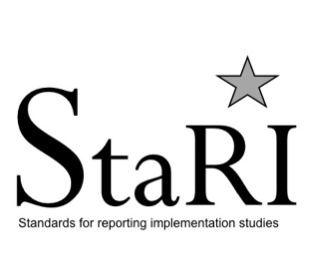
Standards for Reporting Implementation Studies: the StaRI checklist for completion**

The StaRI standard should be referenced as: Pinnock H, Barwick M, Carpenter C, Eldridge S, Grandes G, Griffiths CJ, Rycroft-Malone J, Meissner P, Murray E, Patel A, Sheikh A, Taylor SJC for the StaRI Group. Standards for Reporting Implementation Studies [(StaRI) statement](http://www.bmj.com/content/356/bmj.i6795.full). *BMJ* 2017;356:i6795

| **Checklist item** | | **Reported on page #** | **Implementation Strategy** | **Reported on page #** | **Intervention** |
| --- | --- | --- | --- | --- | --- |
|  | |  | “Implementation strategy” refers to how the intervention was implemented |  | “Intervention” refers to the healthcare or public health intervention that is being implemented. |
| **Title and abstract** | | | | | |
| Title | **1** | 1 | Implementation analysis of patient reported outcomes (PROs) in oncological routine care – an observational study protocol | | |
| Abstract | **2** | 2 | **Background**  The successful implementation of patient-reported outcomes (PROs) in clinical routine faces many challenges, first and foremost the lack of consideration thereof in the patient care process. The aim of this study will be to first identify relevant barriers and facilitators and then design suitable implementation strategies which will be evaluated to improve the effectiveness of a PRO measure assessment in inpatient and outpatient cancer routine care.  **Methods**  During the preparation phase, interviews with oncological patients (N=28) and medical staff (N=4) as well as focus groups with medical staff (N=18) across five different departments caring for cancer patients were conducted. On the basis of these, qualitative content analysis revealed relevant barriers and facilitators for implementation of PROs in cancer care. Subsequently, implementation strategies and a model of implementation were developed. In the study phase, implementation strategies will be evaluated based on nine different implementation outcomes in five different oncological clinics. Evaluation of the implementation process will take place during three months in each clinic and data will be conducted pre, while and post implementation of the PRO measure. Therefore a sample size of 60 participants of whom 30 staff members and 30 participants will be questioned using existing and newly developed implementation outcome evaluation instruments.  **Discussion**  Key to improving the effectiveness of PRO assessment in the time-critical clinical environment is the utilization of easy-to-use, electronic PRO questionnaires directly linked to patients’ records thereby improving consideration of PROs in patient care. In order to validate the effectiveness of this implementation process further, an evaluation parallel to implementation following an observational study design with a mixed-methods approach will be conducted. This study could contribute to the development of adequate evaluation processes of implementation of PROs to foster sustainable integration of PRO measures into routine cancer care. | | |
| **Introduction** | | | | | |
| Introduction | **3** | 3 | As cancer patients often experience physical and psychosocial consequences of their disease and its treatment, evaluation of health-related quality of life (HrQoL) by using Patient Reported Outcomes (PROs) is important to get a full understanding of patient’s needs. It is surprising that the implementation of this information lags behind and that research is required in this respect. Many studies indicate that the use of PROs has been found to be useful, but there is often a lack of clear interpretation and structure for the application of the instrument in clinical routine. Because of many barriers that impact on successful implementation of PROs, it is required to further investigate what facilitates and what hinders implementation to maintain sustainability of PRO interventions in oncological care. Therefore, accurate evaluation of implementation processes is key to foster successful implementation. | | |
| Rationale | **4** | 3-5 | To ensure sustainable use of the PRO measure and its outcomes, the implementation will be evaluated following the dimensions proposed by Proctor et al. (2011) as well as the Consolidated Framework for Implementation Research (CFIR). Proctor et al. (2011) propose eight dimensions following e.g. the RE-AIM Framework promoted by Glasgow (2007) to evaluate implementation of interventions in health care: Acceptability, Adoption, Appropriateness, Cost, Feasibility, Fidelity, Penetration and Sustainability. Implementation of interventions should be evaluated on these eight dimensions in order to gain precise information on the implementation process and to identify possible barriers. | 3 | The use of PROs in oncological routine care to assess HrQoL in cancer patients can improve health care by assessing relevant symptoms and burden in HrQoL. Especially in chronic diseases PROs play an important role to provide data on the patient experience. |
| Aims and objectives | **5** | 5 | The purpose of our study is to identify beneficial and inhibiting conditions for clinicians and patients to use a PRO measure assessing HrQoL in cancer patients in clinical routine care. On the basis of these findings, the PRO measure will be implemented into oncological routine care in a University Hospital in Germany. The implementation process will be evaluated. | | |
| **Methods: description** | | | | | |
| Design | **6** | 5-7 | To evaluate the implementation of the PRO measure, an observational study with a mixed method design will be conducted. The study consists of two phases (preparation phase and study phase) and combines qualitative and quantitative data in an exploratory mixed methods study design. Evaluation will take place pre, while and post implementation of the PRO measure. | | |
| Context | **7** | 5-7 | The intervention will be implemented into oncological routine care at five inpatient and outpatient clinics in a University Hospital in Germany. By involving medical staff members (i.e. nurses and doctors) as well as patients, potential difficulties may occur throughout the implementation process. Most challenging, we have to integrate the PRO measure into the often stressful daily routine of medical staff members. Furthermore, different circumstances in the five participating in- and outpatient clinics will have an impact on the implementation of the intervention. | | |
| Targeted ‘sites’ | **8** | 7 | Recruitment of patients and staff members at the University Medical Center Hamburg-Eppendorf in cooperation with the II. Medical Clinic and Polyclinic, the Department of Stem Cell Transplantation, the Department of Gynecology, the Department of Radiotherapy, Radiation Oncology and Department of Otolaryngology. | 8 | Potential patients to be questioned will be pointed out by staff. The appointed patients will be asked to participate and to give a written consent and will be interviewed by scientific staff.  Inclusion criteria for patients are inpatient or outpatient cancer treatment in one of the five clinics, sufficient language skills in German and no severe cognitive or verbal impairments in providing information and giving informed consent.  Staff members will be pointed out by contact partners in the five clinics. |
| Description | **9** | 6-7 | Every staff member will receive training courses as well as clear instructions about interpretation frames and handling of the questionnaire. Some action examples will be presented. Moreover, the PRO measure will be kept very short avoiding problems due to lack of time. Another aspect facilitating the implementation process is the direct integration of the collected information throughout the PRO measure into the electronic patient file. | Stated elsewhere | The HELP-5 is PRO measure for HrQoL of cancer patients. With five dimensions of one item each the developed questionnaire is short enough, to not disrupt routine procedures during treatment and is profound enough, to inform clinicians about the patient’s psychosocial well being. The HELP-5 will be presented to patients during treatment, e.g. once a week. The items will be rated electronically along a 11-point Likert scale ranging from 0 “not at all” to 10 “very much”. |
| Sub-groups | **10** | 6-7 | In order to assess relevant barriers and facilitators for implementing a PRO measure, several interviews with oncological patients (N = 28) and oncologists (N = 4) were undertaken in advance. To facilitate further discussion and exchange, five focus groups with oncologists, oncological nurses and psychologists were conducted, where they were asked to name possible barriers and facilitators. The results were presented for discussion to a group of eight experts, consisting of psychooncologists, oncologists, quality of life scientists, staff nurses, representatives of the quality management and a representative of a health insurance. On the grounds of the findings and of current state of research, the implementation process and implementation strategies were determined. In order to assess the comprehensibility and feasibility of the evaluation questionnaires a pilot run will be conducted. Up to five staff members of the University Medical Centre will be asked to give their impressions by using the reading out loud technique. | | |
| **Methods: evaluation** | | | | | |
| Outcomes | **11** | 8-9 | “**Benefit**” will be assessed asking patients while and post implementation with one question about the perceived benefit of the PRO measure for the treatment of the patient. Staff members will be questioned on the “**Benefit**” by one question pre implementation on the expected benefit of the PRO measure and post implementation on the actual benefit for the treatment of patients of the PRO measure.  “**Acceptability**” will be assessed using a German translation of the Acceptability E-Scale while as well as post implementation of the PRO measure.  “**Adoption**” will be assessed pre and post implementation using the German translation of the Organizational Readiness for Implementing Change (ORIC) questionnaire.  “**Appropriateness**” will be assessed while and post implementation using the relevance scale of a German translation of the Workshop Evaluation Form (TCU Weval) questionnaire. | 9 | Our primary outcome of the intervention is the HrQoL, which we assessed using the developed PRO measure Help-5. Routine data can be used to analyse development of HrQoL in patients during treatment.  “**Benefit**” will be assessed asking patients while and post implementation with one question about the perceived benefit of the PRO measure for the treatment of the patient. |
| Process evaluation | **12** | 9 | **“Feasibility”** will be assessed pre and post implementation using the program support scale of the Weval questionnaire.  “**Fidelity**” and “**Penetration**” will be assessed while and post implementation using one question for each dimension in a short survey as well as field notes taken by scientific staff members as well as statistical reports of clinical records. “**Sustainability**” will be assessed while and post implementation using two questions on the use of the PRO measure as well as through statistical reports of clinical records and field notes. | | |
| Economic evaluation | **13** | 9 | “**Cost**” will be assessed pre implementation by one question on the expected time taken to record information and address problems according to the PRO measure and post implementation by one question about the time it actually takes for staff members to record information and address possible problems. | 9 | “**Cost**” will be assessed pre implementation by one question on the expected time taken to record information and address problems according to the PRO measure and post implementation by one question about the time it actually takes for staff members to record information and address possible problems. |
| Sample size | **14** | 7, 10 | Interviews with oncological patients (*N* = 28) and oncologists (*N* = 4) were undertaken. To facilitate further discussion and exchange, five focus groups with oncologists, oncological nurses and psychologists were conducted. Patients as well as clinicians were asked to name possible barriers and facilitators for implementation of the PRO measure. The results were presented to a group of eight experts for discussion.  Power calculations according to sample size calculations by Viechtbauer et al. (2015) for this pilot evaluation suggest N=59 with a confidence level of 95% and a low probability of the problem to occur of π=0.05 in total. We therefore chose a number of N=60 in total, n=12 for every clinic participating which leads to n=6 patients and n=6 staff members of whom n=3 nurses and n=3 doctors in each clinic. | | |
| Analysis | **15** | 9-10 | For quantitative data originated in the study phase, analyses of variance will be computed to compare the three different stages during implementation in the five clinics (SPSS Vers. 25). Missing data will be imputed using the expectation–maximization algorithm. Transformations of data will only be applied, if data structure requires so (i.e. non normality of residuals). | | |
| Sub-group analyses | **16** | 9-10 | Sub-group analyses between the five different clinics will be performed. | | |
| **General** | | | | | |
| Statements | **27** | 11-12 | Ethics approval and consent to participate: This study received approval by ethics committee of the medical association Hamburg. Reference number: PV5636.  Routine data is only used within the hospital following data protection regulations.  Trial registration: This study was registered at Open Science Framework (<https://osf.io/y7xce/>).  Funding: This work is supported by “Innovationsfonds des Gemeinsamen Bundesausschusses” grant number [01VSF16024].  Conflict of interest: The authors declare that they have no competing interests. | | |
